# Role of the major antigenic membrane protein in phytoplasma transmission by two insect vector species

**DOI:** 10.1186/s12866-015-0522-5

**Published:** 2015-09-30

**Authors:** Mahnaz Rashidi, Luciana Galetto, Domenico Bosco, Andrea Bulgarelli, Marta Vallino, Flavio Veratti, Cristina Marzachì

**Affiliations:** Istituto per la Protezione Sostenibile delle Piante, CNR, Torino, Italy; DISAFA, Università degli Studi di Torino, Grugliasco, TO Italy; Present address: University of Idaho, College of Agricultural and Life Sciences, Aberdeen, ID USA

**Keywords:** “*Candidatus* Phytoplasma asteris”, Chrysanthemum yellows phytoplasma, *Macrosteles quadripunctulatus*, *Euscelidius variegatus*, *Chrysanthemum carinatum*

## Abstract

**Background:**

Phytoplasmas are bacterial plant pathogens (class Mollicutes), transmitted by phloem feeding leafhoppers, planthoppers and psyllids in a persistent/propagative manner. Transmission of phytoplasmas is under the control of behavioral, environmental and geographical factors, but molecular interactions between membrane proteins of phytoplasma and vectors may also be involved. The aim of the work was to provide experimental evidence that *in vivo* interaction between phytoplasma antigenic membrane protein (Amp) and vector proteins has a role in the transmission process. In doing so, we also investigated the topology of the interaction at the gut epithelium and at the salivary glands, the two barriers encountered by the phytoplasma during vector colonization.

**Methods:**

Experiments were performed on the ‘*Candidatus* Phytoplasma asteris’ chrysanthemum yellows strain (CYP), and the two leafhopper vectors *Macrosteles quadripunctulatus* Kirschbaum and *Euscelidius variegatus* Kirschbaum.

To specifically address the interaction of CYP Amp at the gut epithelium barrier, insects were artificially fed with media containing either the recombinant phytoplasma protein Amp, or the antibody (A416) or both, and transmission, acquisition and inoculation efficiencies were measured.

An abdominal microinjection protocol was employed to specifically address the interaction of CYP Amp at the salivary gland barrier. Phytoplasma suspension was added with Amp or A416 or both, injected into healthy *E. variegatus* adults and then infection and inoculation efficiencies were measured.

An internalization assay was developed, consisting of dissected salivary glands from healthy *E. variegatus* exposed to phytoplasma suspension alone or together with A416 antibody. The organs were then either observed in confocal microscopy or subjected to DNA extraction and phytoplasma quantification by qPCR, to visualize and quantify possible differences among treatments in localization/presence/number of CYP cells.

**Results:**

Artificial feeding and abdominal microinjection protocols were developed to address the two barriers separately. The *in vivo* interactions between Amp of ‘*Candidatus* Phytoplasma asteris’ Chrysanthemum yellows strain (CYP) and vector proteins were studied by evaluating their effects on phytoplasma transmission by *Euscelidius variegatus* and *Macrosteles quadripunctulatus* leafhoppers. An internalization assay was developed, consisting of dissected salivary glands from healthy *E. variegatus* exposed to phytoplasma suspension alone or together with anti-Amp antibody. To visualize possible differences among treatments in localization/presence of CYP cells, the organs were observed in confocal microscopy. Pre-feeding of *E. variegatus* and *M. quadripunctulatus* on anti-Amp antibody resulted in a significant decrease of acquisition efficiencies in both species. Inoculation efficiency of microinjected *E. variegatus* with CYP suspension and anti-Amp antibody was significantly reduced compared to that of the control with phytoplasma suspension only. The possibility that this was due to reduced infection efficiency or antibody-mediated inhibition of phytoplasma multiplication was ruled out. These results provided the first indirect proof of the role of Amp in the transmission process.

**Conclusion:**

Protocols were developed to assess the *in vivo* role of the phytoplasma native major antigenic membrane protein in two phases of the vector transmission process: movement through the midgut epithelium and colonization of the salivary glands. These methods will be useful also to characterize other phytoplasma-vector combinations. Results indicated for the first time that native CYP Amp is involved *in vivo* in specific crossing of the gut epithelium and salivary gland colonization during early phases of vector infection.

**Electronic supplementary material:**

The online version of this article (doi:10.1186/s12866-015-0522-5) contains supplementary material, which is available to authorized users.

## Background

Phytoplasmas are prokaryotes belonging to the class Mollicutes, a group of wall-less microorganisms phylogenetically related to low G + C content, Gram-positive bacteria, that are associated worldwide to diseases of important crops [1]. Phytoplasmas are mainly transmitted by phloem feeding leafhoppers, planthoppers and psyllids within the Hemiptera [2]. Phytoplasma transmission is persistent and propagative, involving a latent period in the vector during which ingested phytoplasmas actively multiply and pass from the alimentary canal through the midgut into the haemocoel, finally colonizing salivary gland cells before being transmitted to a new host plant. Vectors remain infective for their whole life [3]. Transmission of plant pathogens by insect vectors is a complex biological process involving interactions between plant, insect, and pathogen [4], and insect vector specificity plays a key role in the epidemiology of several vector-pathogens [5]. For phytoplasmas, behavioral, environmental and geographical factors are involved in determining vector specificity (reviewed in [3]), but also molecular interactions between membrane proteins of phytoplasma and vector may be implicated. Indeed, such interactions have been evoked in other plant pathogen-vector combinations. For example, spiroplasma spiralin protein binds to insect glycoproteins and plays a key role in the transmission of *Spiroplasma citri* by mediating its adherence to epithelial cells of insect vector gut or salivary gland [6]. The specific binding of spiroplasma phosphoglycerate kinase to vector actin is crucial for internalization of the bacteria in the insect cells, with a direct effect on spiroplasma transmission *in vivo* [7, 8]. Similarly, cell surface haemagglutinin- like proteins of *Xylella fastidiosa* bind to different glycoproteins during initial adhesion steps in the colonization of its xylem feeder vector [9]. The transmission of vector borne bacteria is a complex biological process, probably due to the elaborate composition of the bacterial membrane proteome, as shown by masking different *X. fastidiosa* epitopes with antibodies raised against whole bacterial cells, gum and afimbrial adhesins [10]. Phytoplasmas lack a cell wall, therefore their plasma membrane is in direct contact with the host cytoplasm. Membrane proteins with hydrophilic domains exposed on the outer part of the cell are good candidate partners for molecular interactions between the mollicute and the insect vector. Three types of non- homologous but highly abundant and immunodominant membrane proteins (IDP) have been identified in phytoplasmas: Amp, IdpA, and Imp [11]. These proteins are highly variable even among closely related strains of different ribosomal groups [12–14] and this variability is higher than that of other adjacent metabolic genes or non-coding sequences. Indeed, evolution under strong positive selection has been demonstrated for Amp and Imp [13, 15, 16]. Putative transmembrane proteins are also encoded by phytoplasma plasmid genes which might have a role in interaction with the insect host [17, 18]. One such transmembrane protein of ‘*Ca.* P. asteris’ onion yellows strain (OYP) is preferentially expressed in the infected vector, and its absence in a non-insect-transmissible mutant isolate has been linked to the loss of transmissibility [19]. Recently, a mollicute adhesin motif, present on a putative transmembrane protein of OYP, was shown to be required for interaction with plant and vector proteins *in vitro* [20].

*In vitro* studies have demonstrated that phytoplasma IDPs may interact with both insect and plant host proteins. In the case of OYP, the formation of a complex between Amp and insect actin microfilaments has been correlated with the phytoplasma transmission capability of leafhoppers, suggesting that the interaction between Amp and insect microfilaments plays a role in phytoplasma transmissibility [21]. Much evidence has also indicated a specific *in vitro* interaction between ‘*Ca.* P. asteris’ chrysanthemum yellows strain (CYP) Amp and several vector proteins, among which myosin, actin and ATP synthase [22]. The latter protein was shown to be present on the microvillar external surface of the gut epithelial cells as well as on the plasma membrane of the salivary gland cells, two crucial organs for the infection process. Moreover, phytoplasma IDPs interact with plant proteins, such as the case of ‘*Ca.* P. mali’ Imp interacting with plant actin . The role of the potential interaction between phytoplasma IDPs and vector proteins in the transmission process has not been addressed so far, probably due to the recalcitrant nature of these plant pathogens for which a recent claim of cultivation [23] has not been further supported.

The aims of this work were to provide the first experimental evidence that *in vivo* interaction between phytoplasma membrane protein and vector proteins has a role in the transmission process and to identify the topology of the interaction at the gut epithelium and/or salivary glands, the two barriers encountered by the phytoplasma during its colonization of the vector. Experiments were performed on the phytoplasma CYP, which is associated with a disease of ornamental plants in north-western Italy, and the two leafhoppers *Macrosteles quadripunctulatus* Kirschbaum and *Euscelidius variegatus* Kirschbaum, the most important and efficient vectors of this pathogen. Protocols for separately addressing the two barriers (gut epithelium and salivary glands) were developed, and the *in vivo* interactions between CYP membrane protein Amp and vector proteins were studied by evaluating their effects on phytoplasma acquisition and inoculation by the two insect vectors.

## Methods

### Phytoplasma, plant and insect species

Chrysanthemum yellows phytoplasma (“*Ca.* P. asteris”, 16SrI-B, CYP) was maintained in daisy, *Chrysanthemum carinatum* Schousboe, by periodic insect transmission using *M. quadripunctulatus* . Healthy *M. quadripunctulatus* and *E. variegatus* were reared on oat (*Avena sativa* (L.)) in climatic chambers with 20-25 °C and photoperiod 16:8 h (light:dark), and periodically checked by PCR to assure the absence of phytoplasmas.

### DNA extraction, PCR and qPCR

Total DNA was extracted from individual leafhopper adults at the end of the inoculation access period (IAP) and from leaf tissues as described [24] and suspended in 100 μL of sterile double-distilled water (ddH_2_O). The same method was used to extract total DNA from batches of three salivary glands dissected from *E. variegatus* subjected to internalization assay, and in this case DNA was suspended in 20 μL ddH_2_O. The presence of CYP was assayed by nested PCR driven with universal [25] and specific primers [26] according to the original protocols in a S1000™ Thermal Cycler (BioRad).

CYP titre in dissected salivary glands of *E. variegatus* was measured by quantitative real time PCR (qPCR), as previously described [24] in a CFX Connect™ Real-Time PCR Detection System (Bio-Rad) supported by the CFX Manager™ Software, version 3.0. CYP titer was expressed as number of CYP cells per nanogram of insect DNA.

### Recombinant phytoplasma proteins and antibody, ELISA

To determine the *in vivo* role of CYP Amp, two recombinant constructs of His-tagged fusion proteins (CYfAmp64-224 and CYfAmp64-651, [14]) and a polyclonal antibody raised in rabbit against the former construct (antibody A416, [14]) were used in artificial feeding and abdominal microinjection assays. The fusion proteins were produced in large scale volumes as described in the original paper [14]. Briefly, the *Escherichia coli* BL21 selected clones, harbouring the expression constructs, were pre-cultured at 37 °C overnight in 20 mL Luria Bertani (LB) with ampicillin (50 μg/mL) and chloramphenicol (35 μg/mL), then diluted into 500 mL of LB (no antibiotics) and allowed to grow to an OD600 of 0.4-0.6. The cells were induced (1 mM isopropyl-thiogalactopyranoside (IPTG) (Applichem)) for additional 20 h, and processed for His-tagged fusion protein purification with 0.6 mL of NiNTA beads (Qiagen) under denaturing conditions, as detailed in the manufacturer’s instruction. Purified proteins were then dialysed against 0.1x PBS buffer (137 mM NaCl, 27 mM KCl, 10 mM Na_2_HPO_4_, 1.8 mM KH_2_PO_4_), freeze-dried and stored at −80 °C until use.

Indirect enzyme-linked immunosorbent assays (ELISA) were performed to check persistence of fusion proteins (i) in insects of both species after artificial feeding for 17, 24 and 30 h (24 h feeding on fusion protein plus 6 h on oat) as well as (ii) in microinjected *E. variegatus* adults immediately after microinjection, or after being fed on oat for 1, 4, 16, 24, 40 h after the injection. To check the persistence of CYPfAmp64-224 in artificially fed or microinjected leafhoppers, single insects were crushed in a 1.5 mL tube with a micropestle in 300 μL of carbonate buffer (15 mM Na_2_CO_3_, 35 mM NaHCO_3_, 0.02 % NaN_3_, pH 9.6) and clarified by low speed centrifugation (5 min at 3000 g) at 4 °C. The supernatant from each insect was loaded (80 μL/well) in triplicate into a PVC microtiter wells and healthy insect clarified extracts were used as negative control. A416 (1:5000, final concentration of 1 μg/mL), and HRP-conjugated goat anti-rabbit antibody (1:12000) (Sigma) were used as primary and secondary antibodies, respectively.

### Artificial feeding experiments

An artificial feeding protocol was developed to specifically address the interaction of CYP Amp at the gut epithelium barrier. Feeding medium composition and acquisition access period (AAP) length for artificial feeding experiments were optimized, and protein persistence in fed insects was also confirmed as described (Additional file 1). Fifth instar healthy nymphs of *E. variegatus* and *M. quadripunctulatus* were starved for 2 h, then artificially fed for 24 h on Medium 2 (5 % sucrose, 10 mM Tris/Cl, 1 mM EDTA, pH 8.0) alone (Control) or added with either 1 mg/mL of CYfAmp64-224, or 0.1 mg/mL A416 antibody, or CYfAmp64-224 (1 mg/mL) + antibody A416 (0.1 mg/mL), or CYfAmp64-651 (1 mg/mL). Negative control cages with no feeding medium were included in the experiments. For each treatment, 10 nymphs were allowed to feed per cage. At the end of artificial feeding, all alive insects were transferred to CYP-infected daisies for 4 h AAP, caged on oat to complete latent period (LP, 33 and 24 days for *E. variegatus* and *M. quadripunctulatus*, respectively), and then single caged on healthy daisy plants for 3 d IAP. Surviving insects were collected and separately stored at −20 °C for successive DNA extraction and diagnostic PCR. Inoculated daisies were drench treated to the soil with 7 mg a. i. per plant (ACTARA, Syngenta), maintained in the greenhouse under controlled conditions (25 °C, photoperiod L16:D8), observed for CYP-specific symptom appearance for one month, and checked by PCR. For each treatment, transmission efficiency (percentage of PCR positive plants following inoculation with all insects), acquisition efficiency (percentage of PCR positive insects at the end of IAP), and inoculation efficiency (percentage of PCR positive plants following inoculation with CYP-infected insects) were calculated.

Each artificial feeding experiment was repeated five times.

### Abdominal microinjection experiments

An abdominal microinjection protocol was employed to specifically address the interaction of CYP Amp at the salivary gland barrier. Phytoplasma suspension for microinjection was prepared by crushing 30 CYP-infected *E. variegatus* in 900 μL ice cold filter sterilized injection buffer (300 mM glycine, 30 mM MgCl_2_, pH 8.0; [27]). The extract was clarified by slow centrifugation (10 min, 800 g), and filtered through 0.45 μm sterile filters. All extraction steps were done at 4 °C. Newly emerged healthy *E. variegatus* adults were anaesthetized by CO_2_ flushing for few seconds just before microinjection. Abdominal microinjection was conducted under a stereomicroscope with a Cell Tram Oil microinjector (Eppendorf), and about 0.2 μL of solution were injected between two abdominal segments. Latent period for abdominal microinjection experiments was optimized and protein persistence in microinjected insects was confirmed as described (Additional file 2). To exclude a generic interfering effect of a non phytoplasma protein in CYP transmission under our microinjection protocol, CYP suspension alone or added with BSA were also tested in preliminary experiments (Additional file 2). To establish the *in vivo* role of CYAmp at the salivary gland level, different microinjection solutions were tested. These solutions contained exogenous proteins at the same concentration as those of the artificial feeding experiments: CYP suspension alone or added CYfAmp64-224 (1 mg/mL), antibody A416 raised against CYfAmp64-224 (0.1 mg/mL), CYfAmp64-224 together with A416 antibody (1 mg/mL, and 0.1 mg/mL, respectively) and CYfAmp64-651 (1 mg/mL). Injected insects were caged on oat for 22 d (LP), and then singly transferred to healthy daisies for 2 d IAP. At the end of IAP, all surviving insects were collected and stored at −20 °C for successive DNA extraction, diagnostic and quantitative PCR. Inoculated daisies were treated as described in artificial feeding, maintained in the greenhouse for appearance of CYP-specific symptoms for one month, and checked by PCR. Infection efficiency (percentage of PCR positive insects at the end of IAP), and inoculation efficiency (percentage of PCR positive plants following inoculation with CYP-infected insects) were determined. Each abdominal microinjection experiment with different solutions was repeated twice. Insects microinjected with CYP suspension alone or added with A416 were then classified as ‘Transmitter’ or ‘Non-transmitter’ according to the presence of CYP in the corresponding inoculated plant, and the two groups were considered for successive growth inhibition assay.

### Growth inhibition assay

To exclude that the presence of antibody A416 might inhibit phytoplasma growth and therefore affect inoculation efficiency, a growth inhibition test was adapted from [28, 29]: phytoplasma titer was measured by qPCR in 9 ‘Transmitter’ *E. variegatus* microinjected with CYP suspension only and in 9 ‘Non-transmitter’ individuals following microinjection with CYP suspension plus antibody A416.

### Phytoplasma internalization assay

Salivary glands were dissected from about 150 CO_2_-anesthetized healthy *E. variegatus* adults, kept in 200 μL ice-cold injection buffer and then incubated for 1 h at 4 °C in blocking buffer (1 % BSA in injection buffer). The blocking buffer was then substituted with a fresh CYP suspension (200 μL) obtained as described above for microinjection, by crushing 20 infected *M. quadripunctulatus* individuals (collected at 21 days post acquisition) in blocking buffer alone or added with 1:50 dilution of A416 antibody, for 4 h incubation at 4 °C. As negative controls, 5 or 10 dissected glands were incubated with suspension obtained by crushing 20 healthy *M. quadripunctulatus* individuals alone or added with 1:50 dilution of A416 antibody, respectively. Following 5 washes (200 μL) with injection buffer, 35 organs were fixed for immunofluorescence observations, while the remaining 75 were divided into batches of three and stored at −20 °C for successive DNA extraction, diagnosis and qPCR.

### Immunofluorescence

Midguts excised from *M. quadripunctulatus* adults as well as salivary glands dissected from *E. variegatus* of internalization assays were fixed in 4 % paraformaldehyde, 0.1 M phosphate buffer, pH 7.4, 0.1 % Triton X-100 overnight at 4 °C. Organs were washed three times in phosphate-buffered saline, pH 7.4 (PBS) and permeabilised overnight at 4 °C with PBS and 1 % Triton X-100. Confocal microscopy was performed on 100 μm thick sections, made with a Leica Vibratome model from 8 % agarose embedded organs. Sections were blocked in PBS containing 1 % BSA for 30 min before incubation with appropriate antibodies. Labeling of A416 antibody acquired during 24 h artificial feeding of *M. quadripunctulatus* was obtained by overnight incubation of midgut section with a 1:80 dilution of a FITC conjugated GAR antibody (F1262, Sigma-Aldrich), and subsequent five washes in PBS. Labeling of CYP acquired by *M. quadripunctulatus* following 4 h feeding on infected plants and of *E. variegatus* salivary gland sections from internalization assays was obtained by overnight incubation with A416 antibody (diluted 1:200 or 1:50, respectively), followed by three washes with PBS, further blocking (1 % BSA in PBS, 30 min), incubation for 2 h with a 1:80 dilution of a FITC conjugated GAR antibody (F1262, Sigma-Aldrich), and final washes in PBS (five times). A sequential labelling protocol was employed on midgut sections of *M. quadripunctulatus* to double label A416 antibody and CYP acquired during artificial feeding (24 h) followed by 4 h on infected plants. In this case, labeling of acquired A416 was obtained with 1:80 dilution of an Alexa 633 conjugated secondary antibody (A-21071, Molecular Probes, Invitrogen); after 5 washes in PBS, sections were incubated as detailed above, to label CYP. Control sections were treated in a similar manner, except that either the primary or the secondary antibodies were omitted from the incubation mixture. Midguts dissected from healthy *M. quadripunctulatus* and *E. variegatus* salivary glands of internalization assay negative controls, labeled with primary and secondary antibodies as detailed above, were used to minimize the background noise.

Samples were mounted on microscope slides and observed with a Leica TCS SP2 confocal microscope, using a 406water-immersion objective (HCX Apo 0.80). Laser bands of 488 nm Ar and a 633 nm He/Ne were used to excite FITC and Alexa 633, respectively.

### Data analyses

SigmaPlot 11 (Systat Software, Inc., San Jose, CA) was used to perform statistical tests. The Chi-square test was used to compare transmission, infection, and inoculation efficiencies under different experimental conditions. To compare phytoplasma titer in *E. variegatus* subjected to different experimental treatments, *t*-test was performed.

## Results and discussion

The mechanisms underlying the interaction between phytoplasmas and vectors is still unknown. However, few *in vitro* studies on ‘*Ca.* P. asteris’ indicate that there may be a molecular basis, involving specific interaction between the phytoplasma antigenic membrane proteins (Amp) and some insect proteins. In this work, we aimed at providing evidences for the involvement of Amp in two crucial phases of the vector transmission process: movement through the midgut epithelim and colonization of the salivary glands. Due to the unculturable nature of these plant pathogens [30], this was not an easy task, therefore a large part of the work was dedicated to develop suitable protocols for analyzing the two barriers separately (see Additional files 1 and 2).

### Involvement of CYP Amp in crossing the gut epithelium barrier

To specifically address the interaction of CYP Amp at the gut epithelium barrier, insects were artificially fed with media containing either the recombinant phytoplasma proteins, or the antibody or both. The optimization of the artificial feeding protocol is described in the Additional file 1.

Three parameters were measured to evaluate the *in vivo* effect of pre-feeding on the different proteins: i) transmission efficiency (percentage of PCR positive plants following inoculation with all insects), ii) acquisition efficiency (percentage of PCR positive insects at the end of IAP), iii) inoculation efficiency (percentage of PCR positive plants following inoculation with CYP-infected insects).

Artificial feeding experiments were performed on both *M. quadripunctulatus* and *E.variegatus.* Results from the five repetitions were pooled together as acquisition efficiencies in the Control treatment were similar.

Transmission efficiencies of *M. quadripunctulatus* significantly differed among the five treatments (Chi-square = 15.822, *P* = 0.003, Table 1). In particular, feeding on antibody A416 (raised against CYfAmp64-224) significantly reduced transmission efficiency compared to the Control treatment (Chi-square = 6.914, *P* = 0.009, Table 1). A slight reduction was also observed upon feeding of *M. quadripunctulatus* on antibody A416 added with CYfAmp64-224, although the difference was not significant. In the case of *E. variegatus*, the reduced transmission efficiencies observed upon feeding on A416 (alone or added with CYfAmp64-224) were not significant (Table 1).

**Table 1:** Chrysanthemum yellows phytoplasma transmission efficiencies following artificial feeding experiments

Treatment	*Macrosteles quadripunctulatus*		*Euscelidius variegatus*	
	Initial number of insects	Transmission efficiency	Initial number of insects	Transmission efficiency
Control	165	33.3 %^ac^ (93)	299	23.6 % (106)
CYfAmp64-224	120	36.7 %^ac^ (49)	224	18.2 % (33)
Antibody A416	100	6.7 %^b^ (30)	100	16.3 % (43)
CYfAmp64-224 + Antibody A416	120	25.5 %^ab^ (47)	224	0.0 % (15)
CYfAmp64-651	90	46.0 %^c^ (63)	130	50.0 % (6)

Chrysanthemum yellows phytoplasma (CYP) transmission efficiencies of *Macrosteles quadripunctulatus* and *Euscelidius variegatus* following artificial feeding in the presence and absence of proteins and successive acquisition on CYP-infected plant. Transmission efficiency: percentage of CYP PCR positive plants following inoculation with all insects surviving the latent period. Within columns, figures followed by different letters differ significantly (Chi square test). Sample sizes in parenthesis

The transmission process was further analyzed by measuring acquisition and inoculation efficiencies. Acquisition efficiencies of the Control treatments were 44 % and 52 % for *E. variegatus* and *M. quadripunctulatus* (Table 2), respectively. Higher efficiencies are reported for these species following longer AAP [31], but, under our experimental conditions, suboptimal AAP lengths were required to maximize co-presence of exogenous proteins and phytoplasmas, and to minimize protein degradation. Significant differences were recorded in phytoplasma acquisition by *M. quadripunctulatus* under the different experimental conditions (Chi-square = 33.178, *P* < 0.001, Table 2). Feeding on antibody A416 alone or in combination with CYfAmp64-224 significantly decreased the acquisition efficiency of *M. quadripunctulatus* compared to the Control (Chi-square = 15.761, *P* < 0.001 and Chi-square = 7.611, *P* = 0.006, respectively), while no effects were recorded after artificial feeding on CYfAmp64-224 or CYfAmp64-651 (Table 2). Similarly, acquisition efficiencies of *E. variegatus* significantly decreased only following feeding on antibody A416 alone (Chi-square = 4.478, *P* = 0.034) and combined with CYfAmp64-224 (Chi-square = 13.754, *P* < 0.001) (Table 2). When only CYP-infected *M. quadripunctulatus* and *E. variegatus* were considered, similar inoculation efficiencies were recorded for each species following the different treatments (Table 2).

**Table 2:** Chrysanthemum yellows phytoplasma acquisition and inoculation efficiencies following artificial feeding

Treatment	*Macrosteles quadripunctulatus*		*Euscelidius variegatus*	
	Acquisition efficiency	Inoculation efficiency	Acquisition efficiency	Inoculation efficiency
Control	51.9 %^a^ (106)	62.0 % (50)	44.2 %^a^ (120)	51.0 % (49)
CYfAmp64-224	46.6 %^ac^ (58)	72.0 % (25)	33.3 %^ab^ (45)	54.5 % (11)
Antibody A416	15.6 %^b^ (45)	50.0 % (4)	26.3 %^bc^ (57)	53.8 % (13)
CYfAmp64-224 + Antibody A416	28.1 %^bc^ (57)	92.3 % (13)	11.4 %^c^ (44)	0.0 % (4)
CYfAmp64-651	63.5 %^a^ (63)	72.5 % (40)	33.3 %^abc^ (15)	75.0 % (4)

Acquisition and inoculation efficiencies of chrysanthemum yellows phytoplasma (CYP) by *Macrosteles quadripunctulatus* and *Euscelidius variegatus* following artificial feeding in the presence and absence of proteins, and successive acquisition on CYP-infected plant. Acquisition efficiency: percentage of CYP-infected insects (PCR positive). Inoculation efficiency: percentage of CYP PCR positive plants following inoculation with CYP-infected insects. Within columns, figures followed by different letters differ significantly (Chi square test). Sample sizes in parenthesis

Overall, pre-feeding of *M. quadripunctulatus* and *E. variegatus* on antibody A416, raised against CYfAmp64-224, resulted in a significant decrease of acquisition efficiencies in both species.

**Fig. 1:**
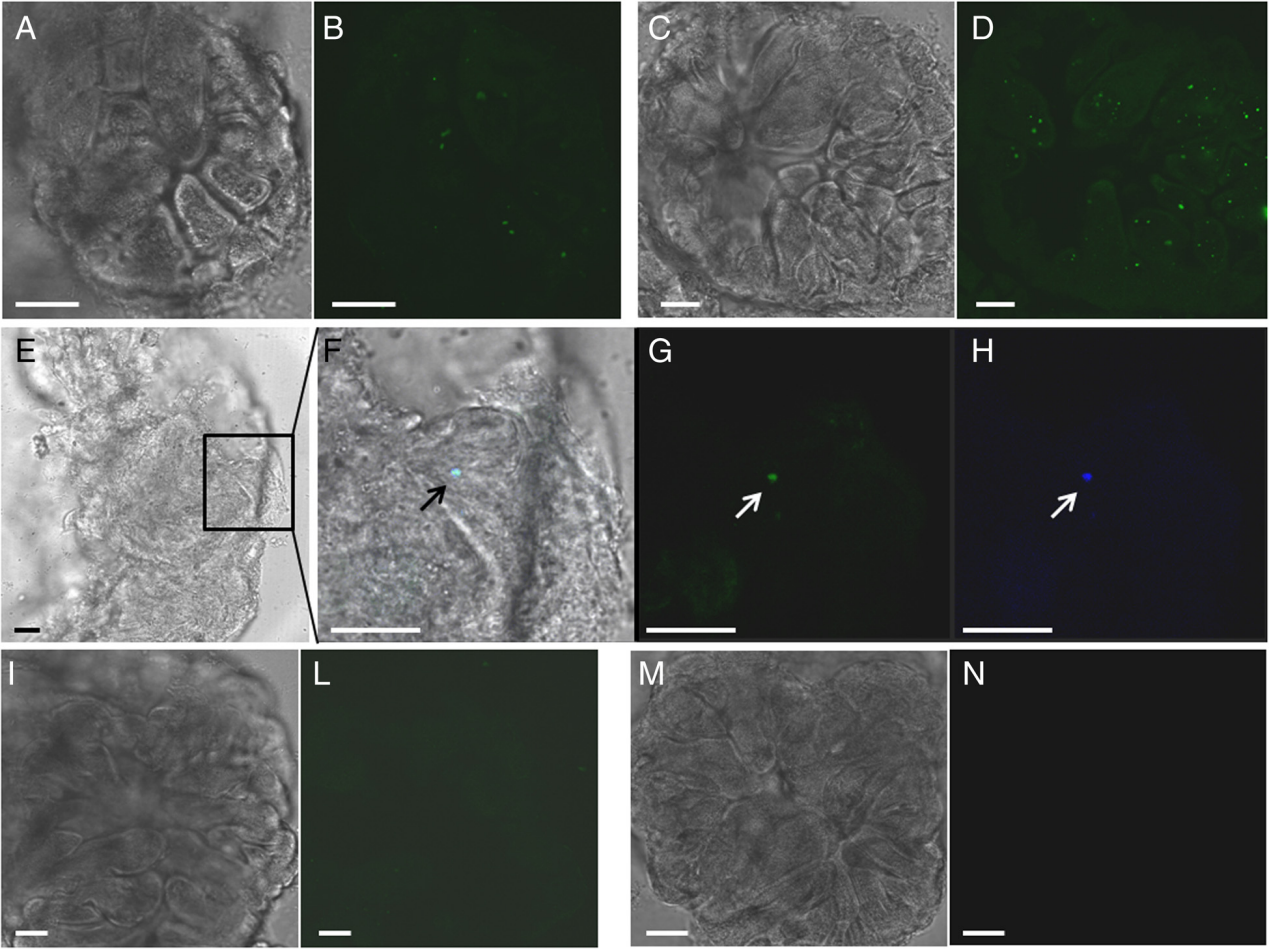
Immunofluorescence of A416 antibody and chrysanthemum yellows phytoplasma (CYP) in vibratome sections of *Macrosteles quadripunctulatus* midguts. In **a** and **b**, labeling of CYP cells after 4 h insect feeding on CYP-infected plants. In **c** and **d**, A416 antibody acquired through 24 h artificial feeding was directly labeled with a goat anti rabbit FITC-conjugated antibody. In **e**-**h**, direct immunofluorescence labeling of A416 antibody with a goat anti rabbit Alexa 633-conjugated antibody (**h**) followed by CYP labeling (FITC, **g**) shows the coincidence of the respective signals (**f**) in insects pre-fed on A416 antibody (24 h) and then on CYP-infected plant (4 h). **i**, **l** and **m**, **n** are negative controls in which primary and secondary antibody (respectively) was omitted. **a**, **c**, **e**, **i**, **m** show bright-field images; **b**, **d**, **g**, **h**, **l**, **n** show fluorescence images; **f** is the overlay of light and fluorescence images. Bars represent 20 μm

Confocal microscopy observations were applied to evidentiate, at the gut level, the co-presence of proteins administered during the pre-feeding and phytoplasma cells acquired from infected plants. A416 antibody acquired through 24 h artificial feeding, directly labeled by the goat anti rabbit FITC-conjugated antibody, was visible in vibratome sections of *M. quadripunctulatus* midguts (Fig. 1d). Also CYP cells showed an intracellular localization in vibratome sections of *M. quadripunctulatus* midguts dissected after 4 h feeding on CYP-infected plant (Fig. 1b). A coincidence of the two signals was evident in midgut sections of *M. quadripunctulatus* (Fig. 1f) fed on A416 antibody (24 h) and on CYP-infected plant (4 h) following direct immunofluorescence labeling of A416 antibody with goat anti rabbit Alexa 633-conjugated antibody (Fig. 1h) and successive CYP labeling (Fig. 1g). Therefore, both A416 and CYP were present in the gut cells at early stages following artificial feeding and infective nutrition, and the co-localization of the specific signals showed that they could interact. These observations can be explained by the specific recognition between antibody A416 and its antigen, the CYP native Amp protein [14]. The masked native Amp would not be able to further interact with putative insect receptors, such as ATP synthase (on plasma membrane of microvillar epithelial cells in the gut lumen) or actin/ myosin (within the epithelial cells), as indicated by previous *in vitro* studies [21, 22]. In this hypothesis, the phytoplasma cells decorated by antibody A416 would not be able to enter or cross the gut barrier. In line with this, despite the reduction of transmission efficiencies in both species, inoculation by CYP-infected insects was not altered. Phytoplasmas that escaped decoration by antibody A416 would have been able to cross the gut barrier, multiply in the haemocoel, colonize the salivary glands and be inoculated as in the Control treatment. Consistently, pre-feeding on antibody A416 significantly decreased acquisition efficiencies of both species compared to the Control treatments also in the presence of CYfAmp64-224.

These results provided the first indirect evidence of the involvement of native CYP Amp in crossing of the gut epithelium during the early phases of vector infection, although further studies are required to identify the insect protein partners of the interaction as well as the level of the interaction (adhesion/internalization or intracellular movement).

### Involvement of CYP Amp in colonization of salivary glands

To specifically address CYP Amp interaction at the salivary gland barrier, abdominal microinjection and an internalization assay were used.

#### Abdominal microinjection experiments

The abdominal microinjection assay was run only on *E. variegatus*, due to its larger size compared to *M. quadripunctulatus*, and to the availability of previous literature supporting this approach to study vector ability of *E. variegatus* [27, 32]. Two parameters were measured to evaluate the *in vivo* effect of different proteins in the microinjected CYP suspension: i) infection efficiency (percentage of PCR positive insects at the end of IAP), ii) inoculation efficiency (percentage of PCR positive plants following inoculation with CYP-infected insects). Optimization of the abdominal microinjection protocol is described in the Additional file 2. For each treatment, infection efficiencies of two experimental repeats were similar, therefore, the results were pooled together. Infection efficiencies of microinjected *E. variegatus* following different treatments ranged from 94.7 to 100 % (Table 3), indicating that microinjection was always successful in delivering CYP into the insect hemocoel. Test plants inoculated by microinjected *E. variegatus* produced CYP-specific symptoms two to three weeks post microinjection, and the results were confirmed by PCR. Significant differences were recorded in phytoplasma inoculation by microinjected *E. variegatus* under the different experimental conditions (Chi-square = 13.790, *P* = 0.008, Table 3). In particular, inoculation efficiency of microinjected *E.variegatus* with antibody A416 was significantly reduced compared to that of the Control (Chi-square = 9.370, *P* = 0.002), while inoculation efficiencies of *E. variegatus* microinjected with CYfAmp64-224 and CYfAmp64-651 were not affected. When insects were microinjected with antibody A416 together with CYfAmp64-224 a slight reduction in inoculation efficiency occurred, although not significant (Table 3).

**Table 3:** Chrysanthemum yellows phytoplasma infection and inoculation efficiencies of *Euscelidius variegatus* following abdominal microinjection

Treatment	Number of microinjected insects	Infection efficiency	Inoculation efficiency
Control	170	99.1 % (117)	56.6 %^a^ (83)
CYfAmp64-224	170	98.9 % (89)	54.5 %^a^ (66)
Antibody A416	186	100 % (72)	26.7 %^b^ (45)
CYfAmp64-224 + Antibody A416	85	95.2 % (62)	38.6 %^ab^ (44)
CYfAmp64-651	81	94.7 % (38)	56.2 %^ab^ (16)

Infection and inoculation efficiencies of *Euscelidius variegatus* following abdominal microinjection with chrysanthemum yellows phytoplasma (CYP) suspension in the presence and absence of proteins. Infection efficiency: percentage of CYP-infected insects (PCR positive) following microinjection. Inoculation efficiency: percentage of CYP PCR positive plants following inoculation with CYP-infected insects. Within columns, figures followed by different letters differ significantly (Chi square test). Sample sizes in parenthesis

Altered inoculation efficiency was not due to a reduced infection efficiency (about 100 % in all the treatments, Table 3), as PCR confirmed that microinjection delivered CYP directly into the insect haemocoel in treatments as well as in the Control. Similarly, the reduced transmission efficiency was not due to a reduced load of phytoplasmas in insects at late stages of the infection cycle. Indeed, in the growth inhibition assay, CYP titer in ‘Non-transmitters’ *E. variegatus* microinjected with phytoplasma suspension plus A416 was similar to that of Control ‘Transmitter’ insects, therefore excluding an inhibition effect of A416 on CYP multiplication (Additional file 3). Moreover, similar phytoplasma titers were reported in *E. variegatus* sampled at late stages of infection [31]. This is in line with previous studies on *E. variegatus* [32] indicating that, despite different transmission ability following microinjection, CYP titer in whole bodies as well as salivary glands were similar between ‘Transmitter’ and ‘Non-transmitter’ individuals.

Taken together, these results suggest a similar scenario as in the artificial feeding assay: the masked native Amp would not be able to further interact with putative insect receptors, and decorated phytoplasma cells would not be able to adhere to, enter or colonize the salivary glands. Some phytoplasmas might escape antibody decoration and therefore be able to continue the infection process, and this would explain why some transmission still occurred after microinjection of CYP together with antibody A416. Consistently, microinjection with antibody A416 plus CYfAmp64-224 slightly decreased transmission efficiency compared to the Control treatment, probably due to competition between native Amp and the fusion antigen for the antibody.

#### Phytoplasma internalization assay

**Fig. 2:**
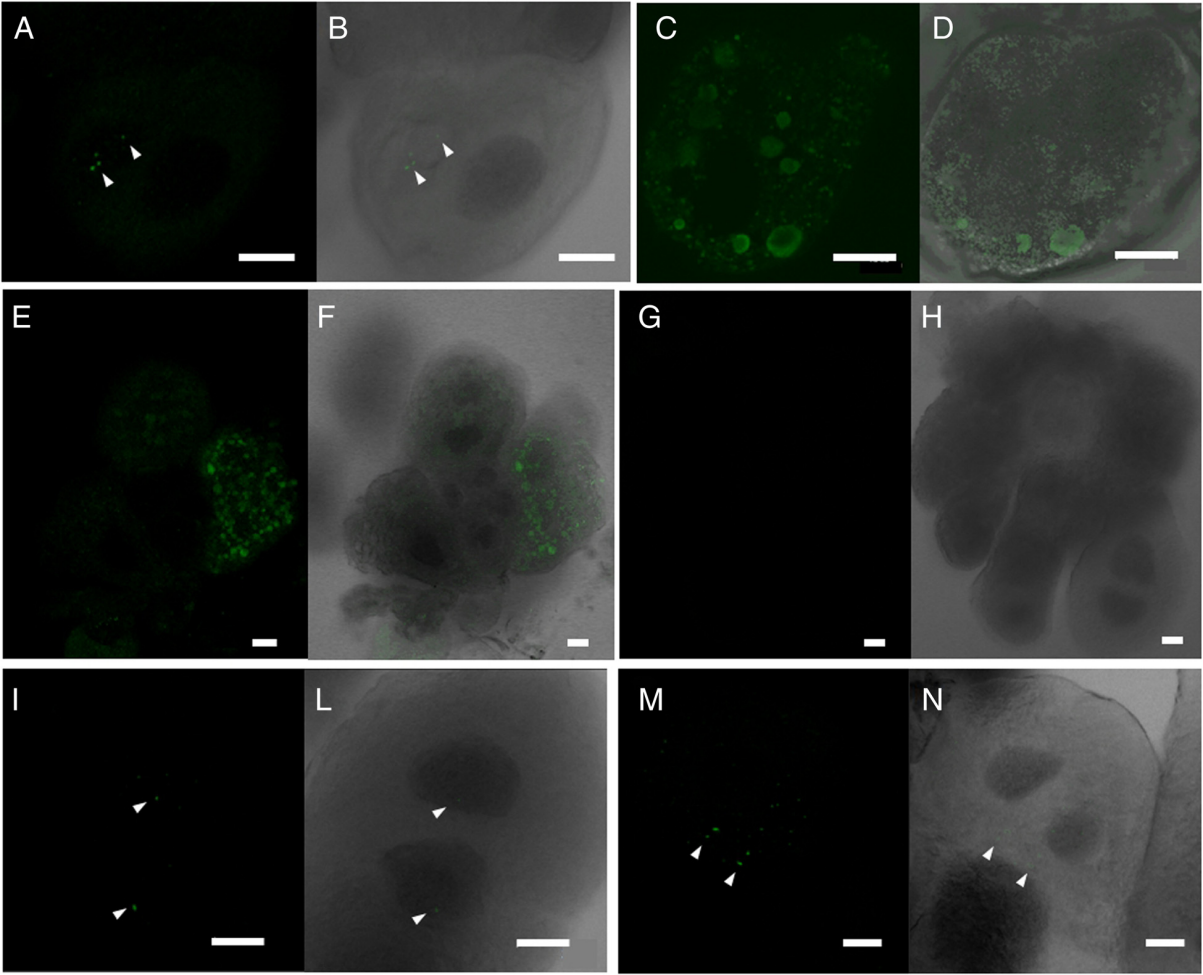
Phytoplasma internalization assay: immunolocalization of chrysanthemum yellows phytoplasma (CYP) in vibratome sections of salivary glands dissected from healthy *Euscelidius variegatus*. In **a**-**f**, salivary glands were incubated for 4 h with an extract of CYP infected *Macrosteles quadripunctulatus*: CYPs are visible as isolated cells (arrowheads in **a** and **b**) and grouped within vesicles (**c**-**f**). In **g** and **h** salivary glands were incubated for 4 h with an extract of healthy *Macrosteles quadripunctulatus* (negative controls). In **i**-**n**, salivary glands were incubated for 4 h with CYP suspension added with A416 antibody: CYPs are visible only as isolated cells (arrowheads). **a**, **c**, **e**, **g**, **i**, **m** show fluorescence images; **b**, **d**, **f**, **h**, **l**, **n** are the corresponding merged light and fluorescence images. Bars represent 20 μm

**Fig. 3:**
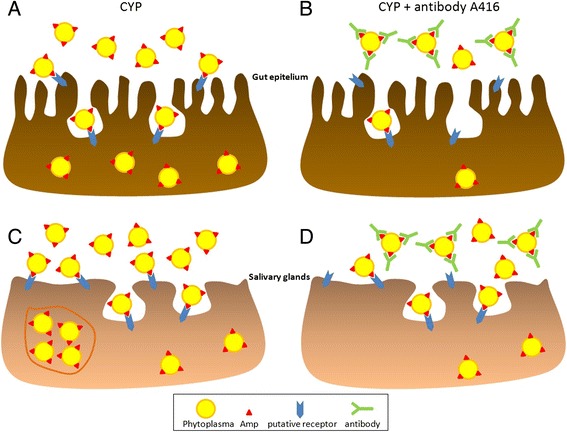
Putative mechanism of phytoplasma adhesion to and internalization in the epithelium of insect vector midgut (**a**, **b**) and salivary glands (**c**, **d**). Following infectious nutrition (**a, b**) or abdominal microinjection (**c**, **d**), phytoplasma cells reach the microvilli of the brush border membrane of vector midgut epithelium (**a**) or the salivary gland epithelium (**c**) of the vector, where native antigenic membrane protein (Amp) molecules within the phytoplasma cell membrane may specifically interact with putative vector receptors, and start vesicle-mediated colonization of host salivary glands (**c**). Masking of native Amp by its antibody A416 (**b**, **d**) impedes the interaction with putative vector receptors, therefore blocking midgut crossing and decreasing acquisition efficiency (**b**) or affecting salivary gland colonization and decreasing inoculation efficiency (**d**)

The reduced transmission efficiency of *E. variegatus*, microinjected with CYP suspension plus A416, is the long-lasting effect of an interaction that presumably occurs at early stages after microinjection. To address this hypothesis, an internalization assay was developed (modified according to [33]), consisting of dissected salivary glands from healthy *E. variegatus* exposed to phytoplasma suspension alone or together with A416 antibody. The organs were then either observed in confocal microscopy or subjected to DNA extraction and phytoplasma quantification by qPCR, to visualize and quantify possible differences among treatments in localization/presence/concentration of CYP cells. This approach was selected to investigate under standardized conditions (organs dissected and incubated in the same amount of CYP cells, for the same time) the very early stages of phytoplasma colonization of salivary glands. This assay enabled us to detect phytoplasma cells in the organs by confocal microscopy as well as qPCR, overcoming the undetectable phytoplasma titer in salivary glands dissected from insects soon after microinjection. Confocal microscopy observation showed that, after 4 h incubation with CYP suspension, CYP cells were visible in two forms: as isolated cells (in four salivary glands out of 15 observed ones; Fig. 2a, b) and packed within vesicles (in two salivary glands out of 15 observed ones; Fig. 2c-f). On the other hand, after 4 h incubation of salivary gland with CYP suspension added with A416 antibody, phytoplasmas were visible only as isolated cells in three salivary glands out of 15 observed organs (Fig. 2i-n). Interestingly, CYP cells packed intracellularly within vesicles were never observed following incubation in the presence of A416. . As negative control, glands were incubated for 4 h with an extract of healthy insects and no signal was detected (Fig. 3g, h). CYP titer in the salivary glands incubated with phytoplasma suspension was very low (less than 100 CYP cells/ng of insect DNA) and similar irrespective of the antibody A416 presence (Additional file 4).

While phytoplasma-packed vesicles were undoubtedly located intracellularly, the cell surface-adhered or free intracellular status of the isolated CYP cells could not be clearly discriminated. In the former hypothesis (cell surface-adhered CYP cells), native Amp would be required for vesicle-mediated internalization of phytoplasmas, and other pathogen membrane proteins would be involved in adhesion to host cells. Indeed, no vesicles were observed when native Amp was masked by A416 antibody. This is also consistent with similar CYP titer detected by qPCR in glands incubated with or without antibody A416, as this approach cannot discriminate the cellular location of the phytoplasma. On the other hand, a free intracellular localization of isolated CYP cells in glands incubated with CYP suspension plus A416 is in line with the reduced, but not abolished, transmission efficiency of this treatment (Table 3). In this case, adhesion and internalization might be mediated by unknown phytoplasma membrane proteins other than Amp, although the possibility that some phytoplasmas might escape antibody decoration and colonize the salivary glands might not be ruled out. In any case, these results provide the first indirect evidence that native Amp is required for vesicle-mediated phytoplasma colonization of vector salivary glands, in an *ex vivo* approach.

## Conclusions

In summary, optimized protocols for *in vivo* assays dissecting the role of gut epithelium and salivary glands in the process of phytoplasma acquisition and inoculation are described here for the first time. These protocols have provided indirect experimental evidences of the *in vivo* role of the phytoplasma native major antigenic membrane protein (Amp) in two phases of the vector transmission process: movement through the midgut epithelium and colonization of the salivary glands (Fig. 3). In fact, our results showed that antibody masking of the native CYP Amp during acquisition feeding resulted in a significant decrease in the acquisition efficiency of both vector species, and also a decrease in transmission efficiency in the case of *M. quadripunctulatus*. Moreover, a significant reduction in inoculation efficiency was also recorded following microinjection into the haemocoel when native CYP Amp was masked. Recombinant CYfAmps administered either by artificial feeding or abdominal microinjection did not alter either acquisition/infection or transmission efficiencies of both species, although the proteins were still detectable by ELISA up to the end of the acquisition access period. This can be explained by several reasons. A cooperative effect of Amp with other phytoplasma membrane proteins or different structural requirements could be necessary for Amp to perform its role. Also, a lower affinity binding could be hypothesized between CYfAmps and putative receptor on the insect cell membrane compared to that of the antibody for its antigen. Degradation of recombinant proteins by insect proteases was excluded, as revealed by ELISA analysis on protein persistence. On the other hand, ten-fold diluted anti-Amp antibody A416 (0.1 mg/mL compared to 1 mg/mL CYfAmps) showed significant effects, therefore suggesting that protein degradation was not a limiting factor.

The protocols described in this work will be extremely useful to characterize other phytoplasma-vector combinations providing unique new tools to prove the involvement of phytoplasma membrane proteins in biologically significant interactions with leafhopper proteins. This is indeed an important issue, and a recent approach has been developed to express phytoplasma membrane proteins at the surface of recombinant spiroplasma cells [34], which will allow future *in vivo* studies of these proteins. Moreover, most of the membrane proteins from phytoplasma genomes available so far have no COG assignment or known function [35], and these assays might contribute to assign a role for some of them in one of the steps of vector colonization. Moreover, the newly developed internalization assay based on dissected salivary glands has been described to further address the very early stages of phytoplasma colonization of these organs under standardized conditions. A similar approach allowed the identification of a phosphoglycerate kinase domain required for internalization of spiroplasma cells in insect cell layers, and the characterization of its effect on *S. citri* transmission [8].

### Availability of supporting data

The data sets supporting the results of this article are included within the article and its Additional files 1, 2, 3, 4.

## Additional files

Additional file 1:
**Optimization of artificial feeding parameters.** Description of parameter optimizations for artificial feeding experiments. (PDF 166 kb) Additional file 2:
**Optimization of abdominal microinjection parameters.** Description of parameter optimizations for abdominal microinjection experiments. (PDF 56 kb) Additional file 3:
**Phytoplasma quantification in ‘Transmitter’ and ‘Non-transmitter’ insects.** Table indicating mean (± standard error) chrysanthemum yellows phytoplasma (CYP) titer measured in *Euscelidius variegatus*, following microinjection with CYP suspension or CYP suspension plus antibody A416. (PDF 282 kb) Additional file 4:
**Phytoplasma quantification in dissected salivary glands.** Table indicating mean (± standard error) chrysanthemum yellows phytoplasma (CYP) titer measured by qPCR in thoroughly washed salivary glands of healthy *Euscelidius variegatus* following incubation with CYP suspension alone or CYP suspension plus antibody A416, according to the protocol developed for the internalization assay. (PDF 178 kb)

## Abbreviations

Amp: Antigenic membrane protein
CYP: Chrysanthemum yellows phytoplasma
CYPfAmp64-224 and CYfAmp64-651: Recombinant constructs of histidine-tagged phytoplasma fusion proteins
A416: Polyclonal antibody raised against CYPfAmp64-224 in rabbit
AAP: Acquisition access period
LP: Latent period
IAP: Inoculation access period
