# Supplementary material for: Role of the major antigenic membrane protein in phytoplasma transmission by two insect vector species
Source: BMC Microbiol. 2015 Sep 30;15:193. doi: 10.1186/s12866-015-0522-5 (PMC4589916; doi:10.1186/s12866-015-0522-5)
Supplement: Additional file 1: — Optimization of artificial feeding parameters. Description of parameter optimizations for artificial feeding experiments. (PDF 166 kb) [file 12866_2015_522_MOESM1_ESM.pdf]

Optimization of artificial feeding parameters

To study *in vivo* interaction between the antigenic membrane protein (Amp) of chrysanthemum yellows phytoplasma (CYP) and vector proteins, preliminary experiments were carried out. Specific protocols were optimized to separately address the two barriers (gut epithelium and salivary glands) encountered by the phytoplasma during colonization of the vector body.

An artificial feeding protocol was developed to specifically address the interaction of CYP Amp at the gut epithelium barrier. Insects were pre-fed on medium containing recombinant CYP Amp or its specific antibody, and then fed on phytoplasma infected plant. Transmission efficiency of single insects was then measured, in order to assess the effect of pre-feeding on different proteins. In particular, feeding medium composition and protein concentrations were optimized to guarantee the best survival of leafhoppers, length of acquisition access period (AAP) was established to maximize co-presence of exogenous proteins and phytoplasmas before protein degradation, and persistence of the recombinant protein was monitored up to the end of the AAP.

Materials and Methods

Feeding medium. To optimize the feeding medium composition, four media (Table A) for *Euscelidius variegatus* and two media for *Macrosteles quadripunctulatus* were tested. Small plastic cages (4 cm diameter) for artificial feeding were covered with ethanol-washed parafilm. About 700 µL of each medium was layered on parafilm, the liquid was covered with new parafilm, and five 5th instar nymphs of each species were transferred to the cage. The cages were kept under constant yellow neon light in a climatic chamber (22°C ± 1°C). Each medium was tested with 5 cages and a control cage devoid of feeding medium was also added. Dead insects were counted at different feeding time and the survival rate on each medium was calculated for *E. variegatus* (Table B), and *M. quadripunctulatus* (Table C). To define the highest protein concentration in relation to survival rate, bovine serum albumin (BSA, Sigma-Aldrich) was added to Medium 2 at concentrations ranging from 0.01 to 1 mg/mL (Table D), and 700 µL of each protein solution was layered on feeding cages. Five 5th instar *E. variegatus* and *M. quadripunctulatus* nymphs from healthy rearing were then transferred into each cage and fed continuously for 48 h. Dead insects were counted, and survival rate for each species was calculated. Optimal antibody A416 concentration was established (0.1 mg/ml) according to adherence inhibition assay of *Mycoplasma agalactiae* to host cells [1], as well as to infectivity neutralization experiments of lettuce infectious yellows Crinivirus on *Bemisia tabaci* [2].

Acquisition access period (AAP). To optimize AAP length, 5th instar nymphs of each species were starved for 2 h, and caged onto CYP-infected daisies for AAP of different lengths. About ten *E. variegatus* and *M. quadripunctulatus* nymphs were removed from the acquisition cage after 1, 2, 4, 6, 8, 13, 17 and 24 h feeding, caged onto healthy oats (one per each AAP length) for 33 days and 24 days, respectively, to complete latency, then singly caged onto healthy daisies for an IAP of 3 days. At the end of IAP, all alive insects were collected and stored at -20°C for successive DNA extraction and PCR analysis. Inoculated

plants were maintained under greenhouse conditions (25°C, photoperiod L16:D8) and observed for phytoplasma symptom appearance every day for one month.

Persistence of the recombinant protein. Indirect enzyme-linked immunosorbent assays (ELISA) were performed to check persistence of fusion proteins in insects of both species after artificial feeding for 17, 24 and 30 h (24 h feeding on fusion protein plus 6h on oat) as described in the main text.

Results and Discussion

Feeding medium. Preliminary experiments suggested that *E. variegatus* is less prone to survive under artificial feeding conditions, so four media were first tested on this species (Table B). The survival rate of *E. variegatus* after feeding for different times on the four media showed that about 60% *E. variegatus* were alive after 48 hour feeding on Medium 2, while the survival rate at the same observation time on Media 1, 3 and 4 were 9%, 3% and 6%, respectively. This could be ascribed to the high concentration of sucrose in Medium 4 (15% compared to 5% on Medium 2), and to the presence of yeast extract in Medium 3. High sucrose concentrations are required for efficient rearing of whiteflies [3] and aphids [4], but not for leafhoppers [5]. The two best performing media (Medium 1 and 2) were then assayed on *M. quadripunctulatus*. The survival rate of *M. quadripunctulatus* was higher on Medium 2 (Table C) and this was then selected for the successive artificial feeding experiments with the two insect vector species.

To determine the highest protein concentration compatible with acceptable mortality, the survival rate of *E. variegatus* upon different feeding times on different concentrations of BSA (from 1 to 0.01 mg/mL) in Medium 2 were measured (Table D). In the absence of feeding medium, almost all *E. variegatus* insects (96.6%) died if the starvation was prolonged beyond 24 h. Therefore the optimal feeding time on the artificial substrate was set to 24 h for successive experiments. After 24 h feeding, more than 70% of *E. variegatus* were alive on 0.5 and 0.05 mg/mL of BSA in Medium 2, and more than 40% of insects were still alive on 1 mg/mL. The survival rate of *M. quadripunctulatus* upon feeding on 1 mg/mL BSA in Medium 2 was over 90% (not shown). One mg/mL protein concentration was then selected for successive artificial feeding experiments, in line with previous competitive binding and internalization experiments of spiroplasmas on insect cell layers [6].

Acquisition access period (AAP). To define the most suitable AAP to guarantee co-presence of exogenous proteins and phytoplasmas before protein degradation, different feeding times on CYP-infected source plant were tested (Table E). For both species, 4 h AAPs were required to successfully transmit CYP and to reach an acquisition efficiency of about 30%, and this time was selected for successive experiments. Different latent periods (LPs, 24 and 33 d) were selected for the artificial feeding experiments of *M. quadripunctulatus* and *E. variegatus,* in line with previous knowledge [7]. Shorter LPs are reported for both species (18 and 30 d for *M. quadripunctulatus* and *E. variegatus*, respectively), but following longer AAPs of 7 d [7].

Persistence of the recombinant protein. Protein persistence was verified by ELISA in artificially fed insects: following 24 h feeding on the fusion protein CYfAmp64-224 (1 mg/mL in Medium 2), the protein was still detectable in both species up to the end of the acquisition access period (AAP, 30 h from the onset of

artificial feeding), enough to compete with CY phytoplasma acquired during the 4 h AAP on infected plant (not shown).

Tables

Table A. Composition of tested feeding media

Compounds	Medium 1	Medium 2	Medium 3	Medium 4
Sucrose	10%	5%	5%	15%
Fructose	0.2%	/	/	/
KH_2PO_4	73 mM	/	/	/
$MgCl_2$	3 mM	/	/	/
Tris/Cl	/	10 mM	10 mM	/
EDTA	/	1 mM	1 mM	/
Yeast extract	/	/	5%	5%
pH	7.2	8	8	6.9

Table B. Survival rate of *Euscelidius variegatus* after artificial feeding for different times on four media.

Feeding time (h)	N° of alive insects/N° of total insects			
	**Medium 1**	**Medium 2**	**Medium 3**	**Medium 4**
0	22/22	50/50	32/32	31/31
14	21/22	47/50	23/32	26/31
17	21/22	47/50	19/32	22/31
23	15/22	41/50	13/32	11/31
39	15/22	39/50	6/32	6/31
42	2/22	32/50	5/32	5/31
48	2/22	31/50	1/32	2/31
64	2/22	19/50	0/32	1/31
86	NT*	12/50	0/32	0/31
158	NT*	6/50	0/32	0/31

* NT: Not tested

Table C. Survival rate of *Macrosteles quadripunctulatus* after artificial feeding for different times on two media.

Feeding time (h)	N° of alive insects/N° of total insects	
	Medium 1	Medium 2
0	19/19	20/20
14	19/19	20/20
17	18/19	20/20
23	18/19	20/20
39	18/19	20/20
42	18/19	20/20
48	15/19	20/20

Table D. Survival rate of *Euscelidius variegatus* after artificial feeding for different times on different BSA concentrations in Medium 2.

Feeding time (h)	N° of alive insects/N° of total insects on Medium 2 with different BSA concentrations (mg/mL)						No medium
	1	0.5	0.1	0.05	0.01	0	
0	49/49	51/51	51/51	24/24	30/31	76/78	30/30
1	49/49	50/50	51/51	24/24	30/31	75/78	30/30
2	49/49	50/51	51/51	24/24	30/31	75/78	30/30
3	42/49	48/51	51/51	24/24	30/31	75/78	29/30
4	42/49	46/51	51/51	23/24	30/31	75/78	27/30
5	41/49	45/51	43/44	23/24	30/31	72/78	23/30
7	37/49	45/51	43/44	23/24	30/31	72/78	21/30
24	20/49	38/51	23/44	17/24	28/31	56/78	1/30
27	11/49	35/51	18/44	17/24	28/31	46/78	1/30
32	7/49	32/51	17/44	16/24	26/31	34/78	0/30
48	4/49	16/51	5/44	12/24	10/31	26/78	0/30

Table E. Optimization of acquisition access period (AAP). Optimization of AAP required by *Euscelidius variegatus* and *Macrosteles quadripunctulatus* to acquire (N° of PCR positive insects/N° of total analysed insects) and transmit chrysanthemum yellows phytoplasma (Pos: Presence of phytoplasma specific symptoms on inoculated test daisy; Neg: symptomless plant).

AAP length (h)	*Euscelidius variegatus*		*Macrosteles quadripunctulatus*	
	**Acquisition**	**Transmission**	**Acquisition**	**Transmission**
1	0/14	Neg	2/5	Neg
2	3/16	Neg	4/5	Neg
4	3/11	Pos	5/11	Pos
6	4/18	Pos	5/9	Pos
8	4/13	Pos	4/9	Pos
13	3/13	Pos	8/10	Pos
17	4/13	Pos	3/4	Pos
24	10/21	Pos	12/16	Pos

References

1. Fleury B, Bergonier D, Berthelot X, Peterhans E, Frey J, Vilei EM: **Characterization of P40, a cytadhesin of Mycoplasma agalactiae**. *Infect Immun* 2002, **70**(10):5612-5621.
2. Tian T, Rubio L, Yeh HH, Crawford B, Falk BW: **Lettuce infectious yellows virus: in vitro acquisition analysis using partially purified virions and the whitefly Bemisia tabaci**. *J Gen Virol* 1999, **80**(5):1111-1117.
3. Davidson EW, Fay ML, Blackmer J, Lavine M: **Improved artificial feeding system for rearing the whitefly Bemisia argentifolii (Homoptera : Aleyrodidae)**. *Fla Entomol* 2000, **83**(4):459-468.
4. Dadd RH, Mittler TE: **Studies on the artificial feeding of the aphid Myzus persicae (Sulzer)--3. Some major nutritional requirements**. *J Insect Physiol* 1965, **11**(6):717-743.
5. Hou RF, Brooks MA: **Continuous rearing of the aster leafhopper, Macrosteles fascifrons, on a chemically defined diet**. *J Insect Physiol* 1975, **21**(8):1481-1483.
6. Labroussaa F, Dubrana MP, Arricau-Bouvery N, Beven L, Saillard C: **Involvement of a Minimal Actin-Binding Region of Spiroplasma citri Phosphoglycerate Kinase in Spiroplasma Transmission by Its Leafhopper Vector**. *PLoS ONE* 2011, **6**(2): e17357.
7. Bosco D, Galetto L, Leoncini P, Saracco P, Raccah B, Marzachi C: **Interrelationships between "Candidatus Phytoplasma asteris" and its leafhopper vectors (Homoptera : Cicadellidae)**. *J Econ Entomol* 2007, **100**(5):1504-1511.
