# Supplementary material for: Role of the major antigenic membrane protein in phytoplasma transmission by two insect vector species
Source: BMC Microbiol. 2015 Sep 30;15:193. doi: 10.1186/s12866-015-0522-5 (PMC4589916; doi:10.1186/s12866-015-0522-5)
Supplement: Additional file 2: — Optimization of abdominal microinjection parameters. Description of parameter optimizations for abdominal microinjection experiments. (PDF 56 kb) [file 12866_2015_522_MOESM2_ESM.pdf]

Optimization of abdominal microinjection parameters

To study *in vivo* interaction between the antigenic membrane protein (Amp) of chrysanthemum yellows phytoplasma (CYP) and vector proteins, preliminary experiments were carried out. Specific protocols were optimized to separately address the two barriers (gut epithelium and salivary glands) encountered by the phytoplasma during colonization of the vector body.

An abdominal microinjection protocol was developed to address CYP Amp interaction at the salivary gland barrier. Insects were directly injected with a phytoplasma suspension alone or added with CYP Amp or its specific antibody, in order to bypass the gut epithelium barrier. Transmission efficiencies of single injected insects were then measured, in order to assess the effect of different proteins in the injected suspension.

Materials and Methods

Latent period. To determine the minimal latent period (LP), insects injected with CYP suspension were caged on oat for 5 d, and then sequentially transferred to new healthy daisies for 2 d inoculation access period (IAPs) for one month (14 IAPs in total, two exposed plants for each IAP). Exposed daisies (28 plants) were treated as described in artificial feeding, and maintained in the greenhouse for appearance of CYP-specific symptoms for one month.

Persistence of the recombinant protein. Indirect enzyme-linked immunosorbent assays (ELISA) were performed to check persistence of fusion proteins in microinjected *E. variegatus* adults immediately after microinjection, or fed on oat for 1, 4, 16, 24, 40h after the injection as described in the main text.

Effect of BSA as a control protein on survival, infection and inoculation efficiencies. To exclude a generic interfering effect of a non phytoplasma protein in CYP transmission under our microinjection protocol, CYP suspension alone or added with BSA (1 mg/mL) were tested in preliminary experiments. Injected insects were caged on oat for 22 d (LP), and then singly transferred to healthy daisies for 2 d IAP. At the end of IAP, all surviving insects were collected and stored at -20°C for successive DNA extraction and diagnostic PCR. Inoculated daisies were treated as described in artificial feeding, maintained in the greenhouse for appearance of CYP-specific symptoms for one month, and checked by PCR. Infection efficiency (percentage of PCR positive insects at the end of IAP), and inoculation efficiency (percentage of PCR positive plants following inoculation with CYP-infected insects) were determined.

Results and Discussion

Latent period. Symptom observation of plants inoculated by *E. variegatus* at different days post microinjection (dpm) with a CYP suspension was used to set up the optimal LP for successive experiments with different proteins in the injection medium. Test plants showed CYP-specific symptoms from 19 dpm; accordingly, a 21 day LP was selected for successive experiments. This LP was shorter than that optimized for the artificial feeding protocol (21 vs 33): the direct injection of phytoplasmas within the insect haemocoel (therefore avoiding the time required by the bacteria to cross the gut epithelium after AAP on the infected plant) may explain the difference.

Persistence of the recombinant protein. Protein concentrations in the injection medium were the same as those of the artificial feeding protocol. The persistence up to 4 hours post microinjection of CYfAmp64-224 in the microinjected *E. variegatus* was confirmed by ELISA (not shown).

Effect of BSA as a control protein on survival, infection and inoculation efficiencies. Survival rate, infection and inoculation efficiencies of microinjected *E.variegatus* following microinjection of CYP suspension alone or added with BSA were similar (Table A) and, therefore excluding a generic interfering effect of a non phytoplasma protein in CYP transmission under our experimental conditions.

Tables

Table A. Survival rate, infection and inoculation efficiencies of *Euscelidius variegatus* following abdominal microinjection with chrysanthemum yellows phytoplasma (CYP) suspension in the presence and absence of BSA. Survival rate: percentage of live insects at the end of inoculation access period. Infection efficiency: percentage of CYP-infected insects (PCR positive) following microinjection. Inoculation efficiency: percentage of CYP PCR positive plants following inoculation with CYP-infected insects. Sample sizes in parenthesis.

Treatment	Survival rate	Infection efficiency	Inoculation efficiency
Control	49.4% (85)	100% (61)	54.8% (42)
BSA	49.4% (85)	98.2% (56)	58.5% (41)
