# Supplementary material for: Role of the major antigenic membrane protein in phytoplasma transmission by two insect vector species
Source: BMC Microbiol. 2015 Sep 30;15:193. doi: 10.1186/s12866-015-0522-5 (PMC4589916; doi:10.1186/s12866-015-0522-5)
Supplement: Additional file 3: — Phytoplasma quantification in ‘Transmitter’ and ‘Non-transmitter’ insects. Table indicating mean (± standard error) chrysanthemum yellows phytoplasma (CYP) titer measured in Euscelidius variegatus, following microinjection with CYP suspension or CYP suspension plus antibody A416. (PDF 282 kb) [file 12866_2015_522_MOESM3_ESM.pdf]

Phytoplasma quantification in 'Transmitter' and 'Non-transmitter' insects.

Mean (± standard error) chrysanthemum yellows phytoplasma (CYP) titre (CYP cells/ng of insect DNA) measured in *Euscelidius variegatus*, following microinjection with CYP suspension (Control) or CYP suspension plus antibody A416. Insects were collected at the end of the inoculation access period and classified as 'Transmitters' and 'Non-transmitters' according with symptom appearance on corresponding inoculated plants.

Thesis	Insect transmission category	Mean CYP titre ± SE (N)
Control	Transmitters	2.17E+04 ± 5.13E+03 (9)
Antibody A416	Non-transmitters	2.79E+04 ± 2.81E+03 (9)
