# Supplementary material for: Role of the major antigenic membrane protein in phytoplasma transmission by two insect vector species
Source: BMC Microbiol. 2015 Sep 30;15:193. doi: 10.1186/s12866-015-0522-5 (PMC4589916; doi:10.1186/s12866-015-0522-5)
Supplement: Additional file 4: — Phytoplasma quantification in dissected salivary glands. Table indicating mean (± standard error) chrysanthemum yellows phytoplasma (CYP) titer measured by qPCR in thoroughly washed salivary glands of healthy Euscelidius variegatus following incubation with CYP suspension alone or CYP suspension plus antibody A416, according to the protocol developed for the internalization assay. (PDF 178 kb) [file 12866_2015_522_MOESM4_ESM.pdf]

Phytoplasma quantification in dissected salivary glands.

Mean (± standard error) chrysanthemum yellows phytoplasma (CYP) titre (CYP cells/ng of insect DNA) measured by qPCR in thoroughly washed salivary glands of healthy *Euscelidius variegatus* following incubation (4 h) with CYP suspension alone (Control) or CYP suspension plus antibody A416, according to the protocol developed for the internalization assay.

**Thesis**	**Mean ± SE (N)**
Control (CYP)	58.81±13.41 (12)
CYP + Antibody A416	55.81±13.46 (12)
